# Supplementary figures and images for: RNA structures within Venezuelan equine encephalitis virus E1 alter macrophage replication fitness and contribute to viral emergence
Source: PLoS Pathog. 2024 Sep 27;20(9):e1012179. doi: 10.1371/journal.ppat.1012179 (PMC11463830; doi:10.1371/journal.ppat.1012179)

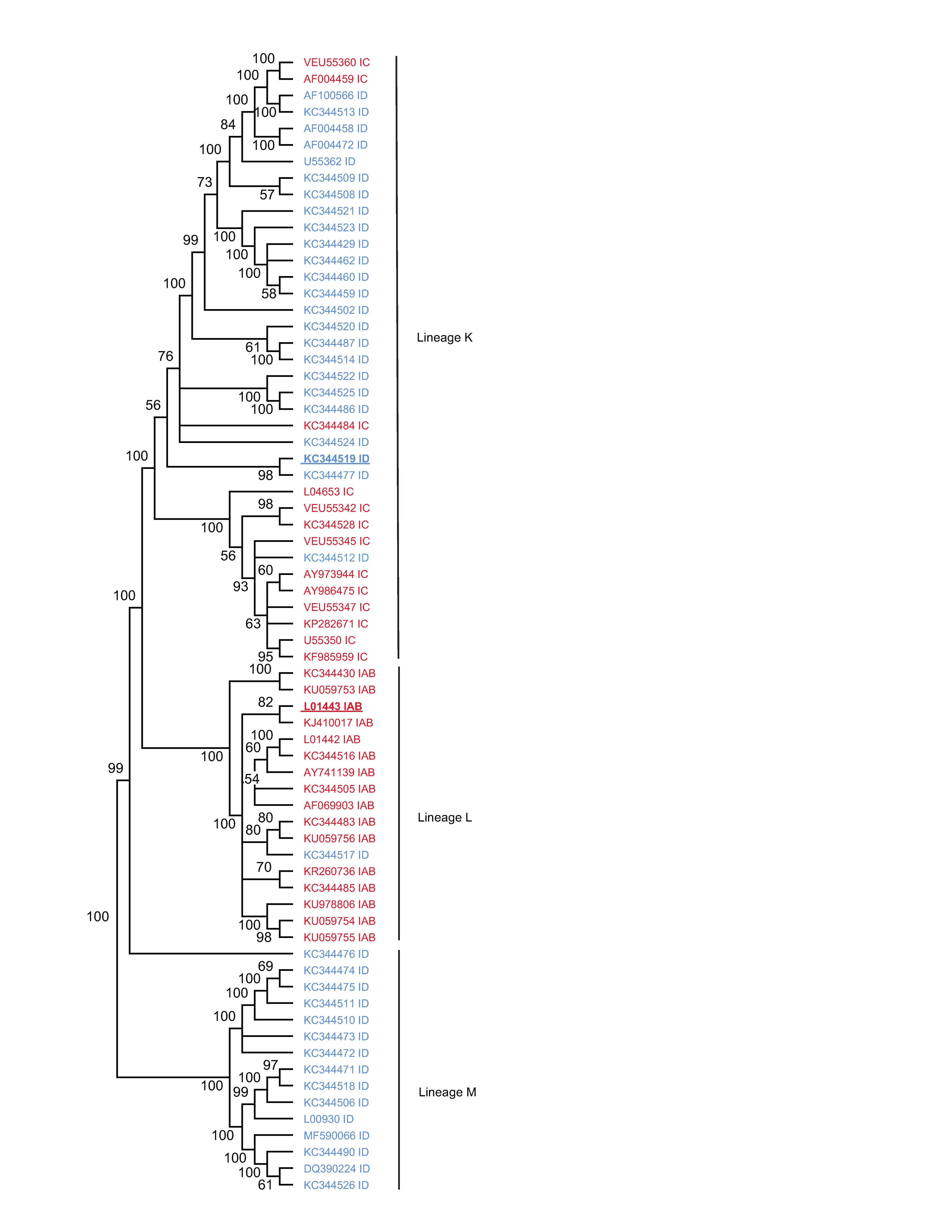

Supplement: S1 Fig — The optimal phylogenetic tree of lineages K, L and M (previously described in [49]) as determined by the neighborhood-joining method [77]. Shown next to each branch is the percentage of replicate trees in which the associated taxa clustered together in the bootstrap test (1000 replicates). (TIF) [file ppat.1012179.s005.tif]

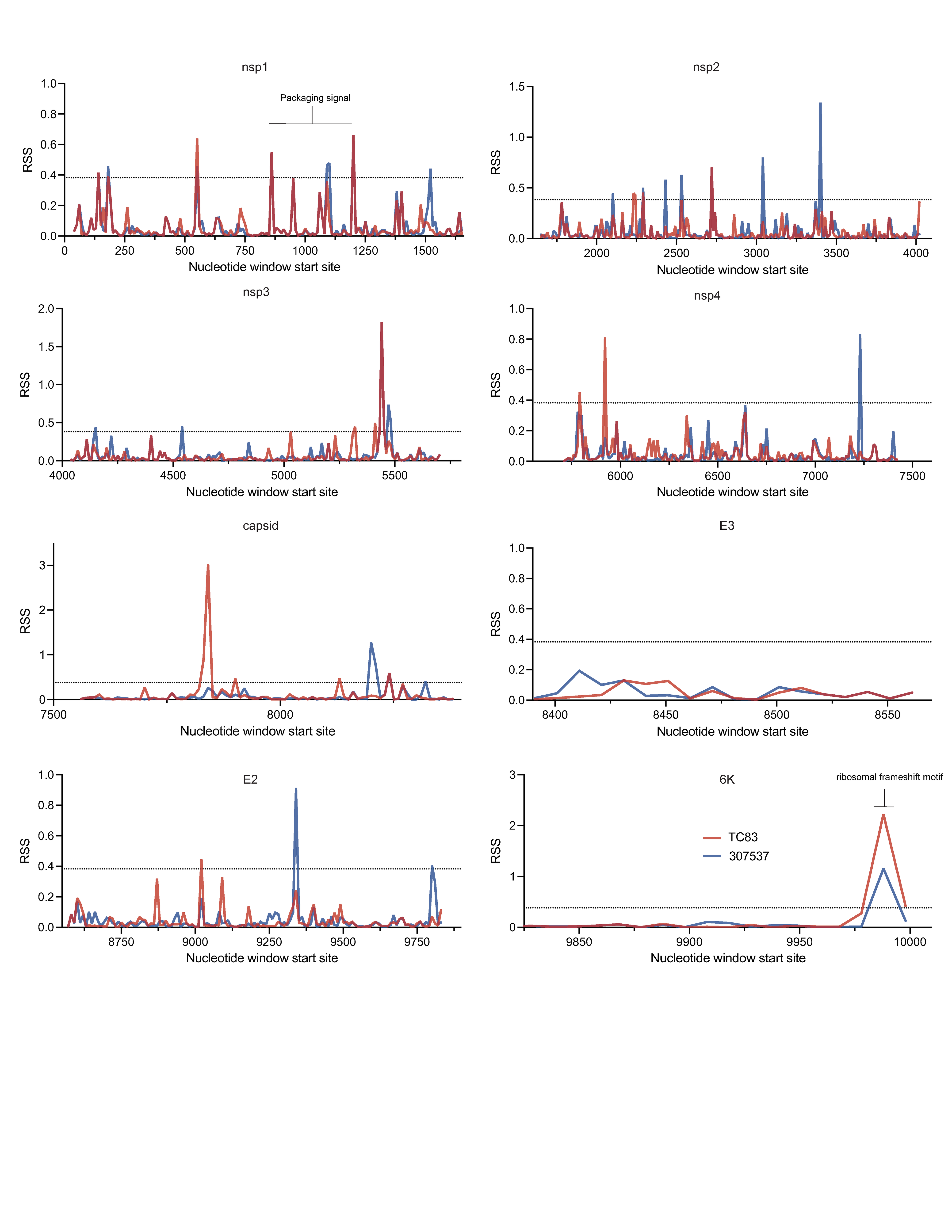

Supplement: S2 Fig — The RSS was calculated as the minimum free energy (MFE)/ensemble diversity for each window of 50 nucleotides with a step size of 10. TC83 is shown in red and 307537 is shown in blue. Two standard deviations from the mean was calculated across the entire genome and is represented as a dotted line. (TIF) [file ppat.1012179.s006.tif]

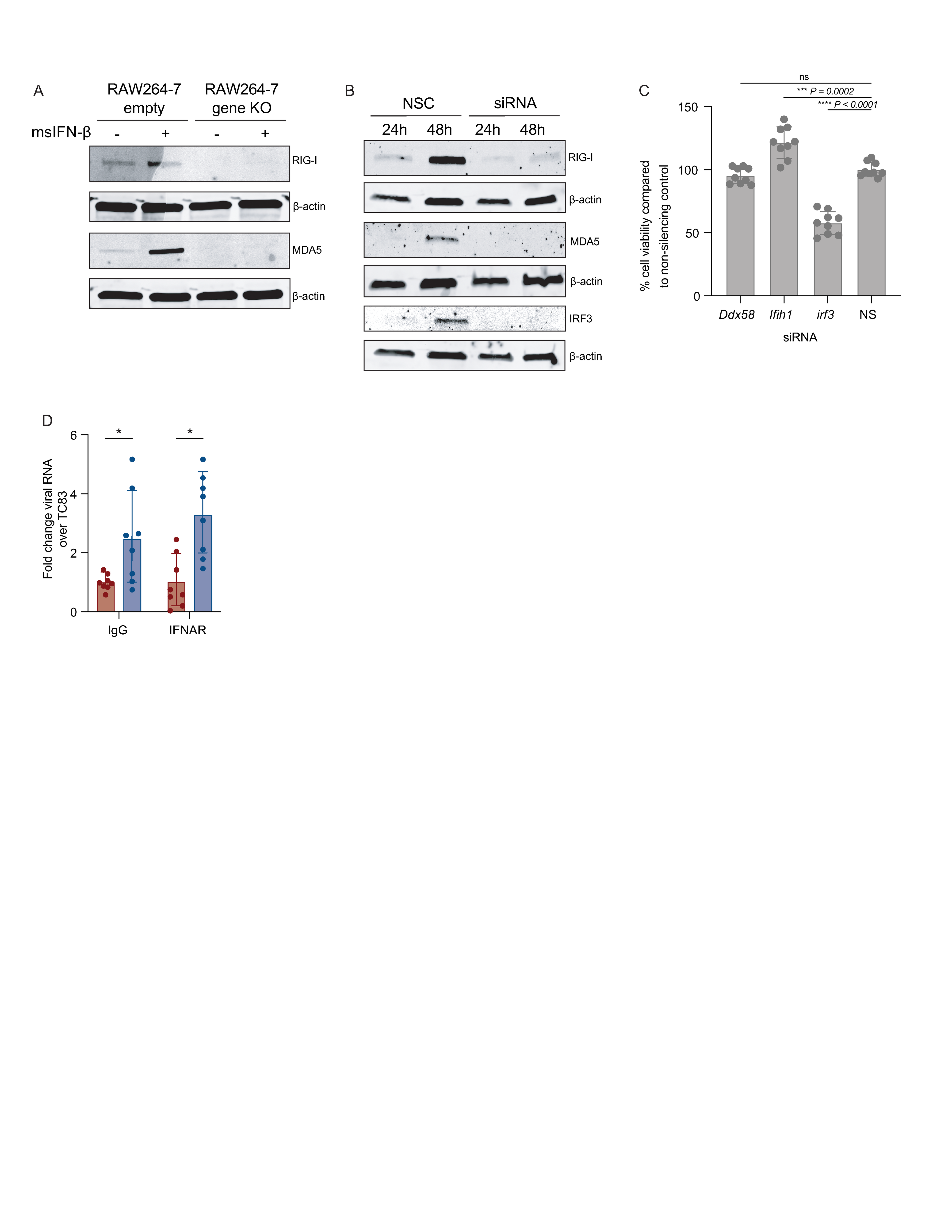

Supplement: S3 Fig — (A) Western blots from Raw264.7 6hrs after treatment +/- 100U/ml msIFN-β. (B) Western blot of Raw264-7 after transfection with NSC or protein of interest siRNA siRNA pool (10μM) pool for 24hrs or 48hrs. (C) Cell viability was determined using alamarblue Cell Viability Reagent and calculated as a percentage compared to the NSC. (D) qPCR was performed for viral RNA and the fold change in viral RNA over TC83 is expressed. Statistical analysis was performed using GraphPad Prism 9, using an unpaired T-test. (TIF) [file ppat.1012179.s007.tif]

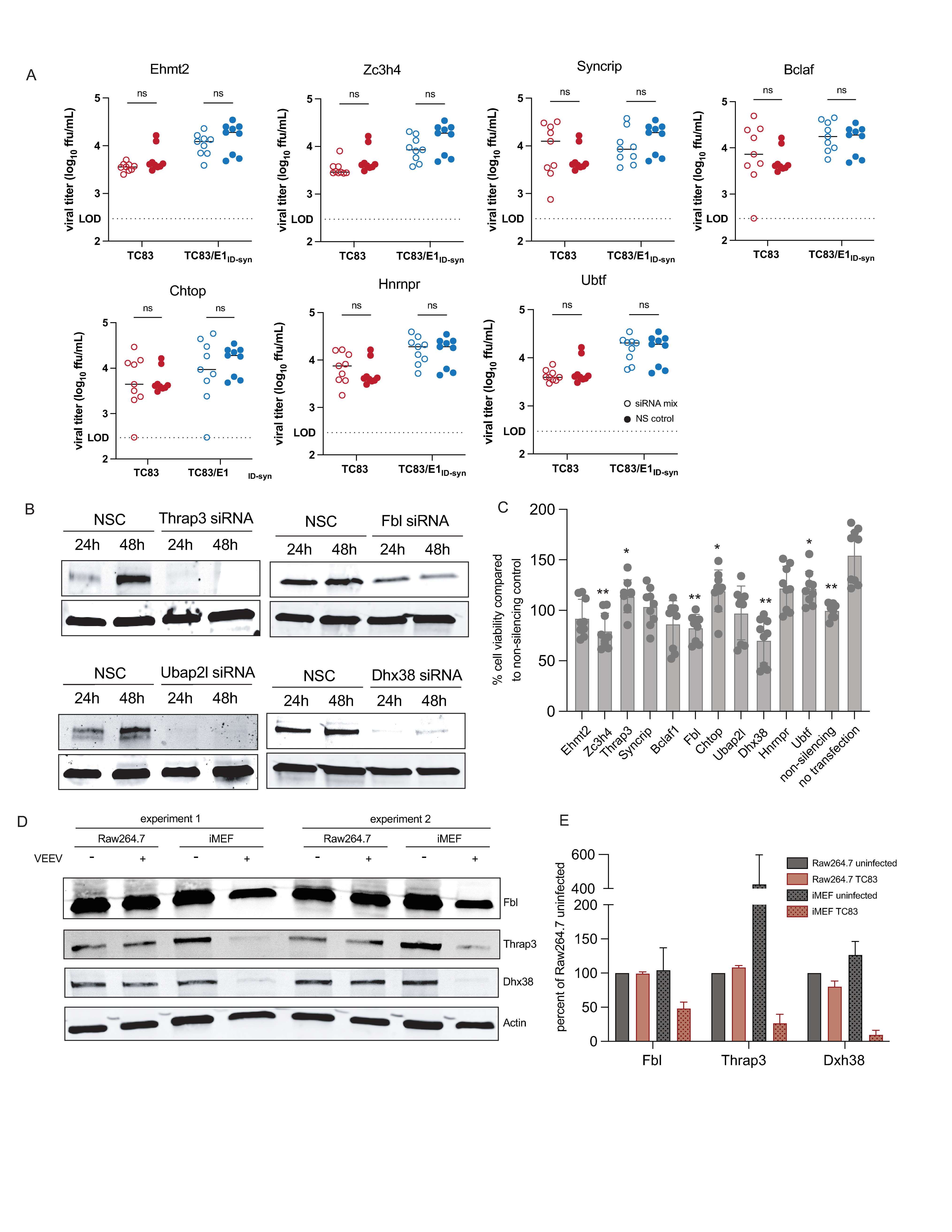

Supplement: S4 Fig — To evaluate the effect of these genes on viral replication (A) Raw264-7 cells were transfected with 10mM of a pool of 3 siRNA targeting proteins of interest for 24hrs, after which they were infected with TC83 or TC83/E1ID-syn. Supernatants were collected at 24hpi and infectious virus was titered using FFA. For visual clarity, the individual siRNAs along with the non-silencing control (NSC) are graphed individually, however the NSC is the same in all graphs. Each experiment was performed in triplicate three times independently and the mean and SD are graphed. (B) Western blot of Raw264-7 transfected for 24h or 48h with NSC or protein of interest siRNA pool (10μM). (C) Cell viability was determined using alamarblue Cell Viability Reagent and calculated as a percentage compared to the NSC. (D) Western blot analysis of Raw264.7 or iMEF +/- TC83 (MOI 0.1) lysates at 24hpi. (E) Densitometry was performed using Adobe Photoshop and expression of proteins were normalized to the actin control. The percentage expression was then calculated in relation to the uninfected Raw264.7 control. This is representative of two independent repeats. Statistical analysis was performed using GraphPad Prism 9, using an unpaired T-test. * >0.05, **>0.001. (TIF) [file ppat.1012179.s008.tif]

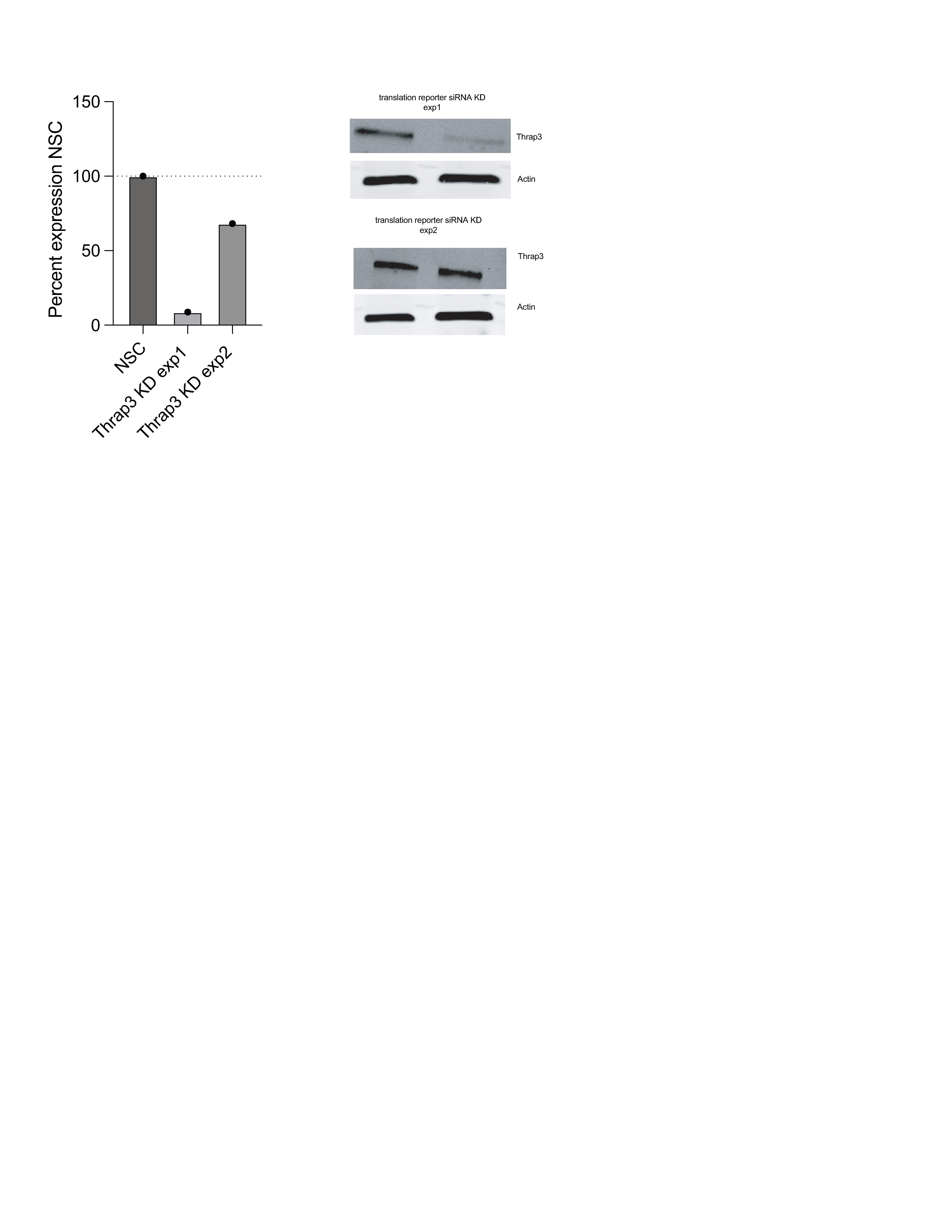

Supplement: S5 Fig — The integrated density of each of the western blot bands was determined using Adobe Photoshop and normalized to the actin control. The expression of each protein is displayed as a percentage of the raw264.7 uninfected control. (TIF) [file ppat.1012179.s009.tif]

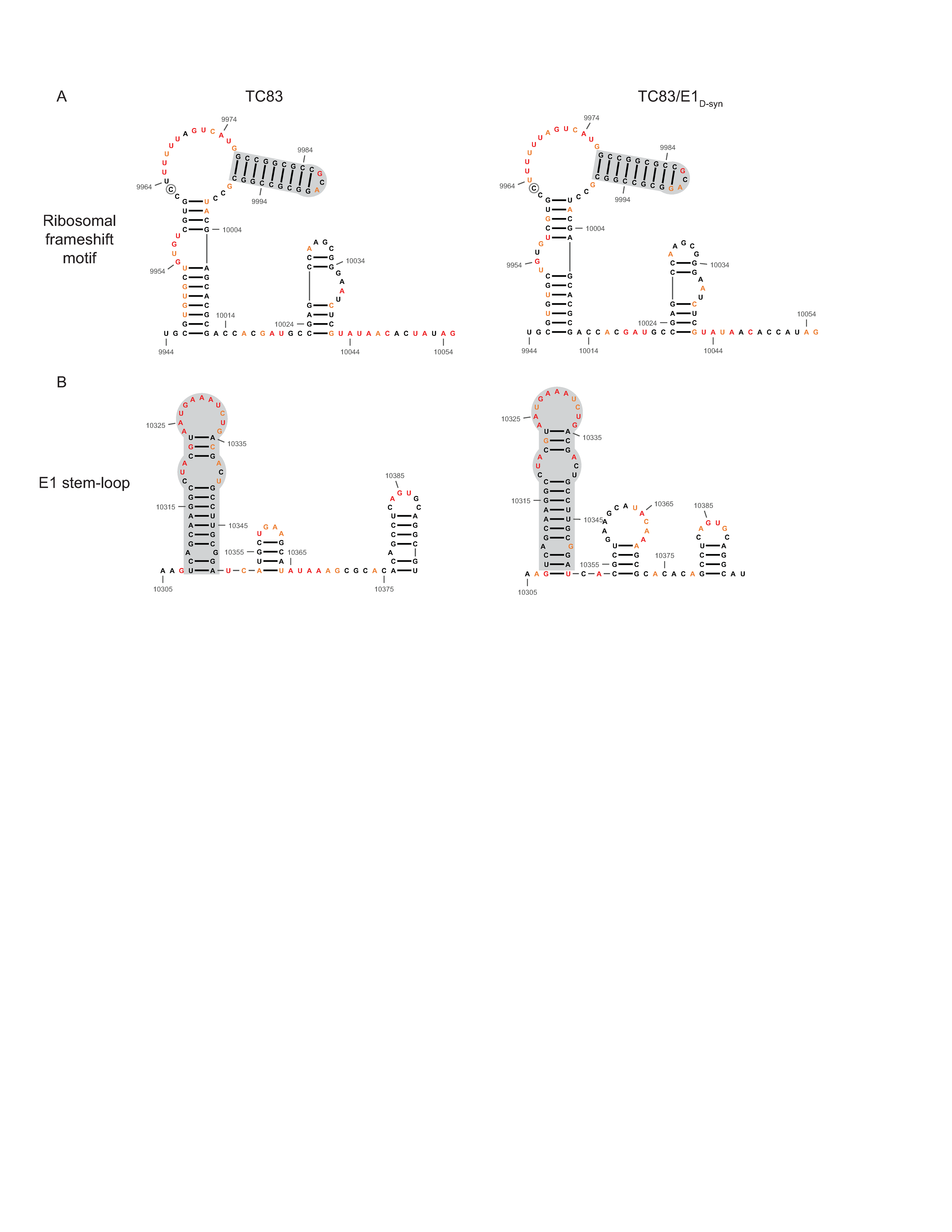

Supplement: S6 Fig — Previous work by Kutchko et al. [47] performed in vitro SHAPE-MaP of the enzootic ID VEEV strain, ZPC738, and identified stable RNA structures across the VEEV genome. Displayed here are the SHAPE-MaP informed secondary structure predictions of (A) the ribosomal frameshift motif and (B) an E1 stem-loop for TC83 and TC83/E1ID-syn. Shaded in grey are the conserved regions identified between the previously described stable structures in ZPC738 and the in vivo SHAPE-MaP data generated for TC83 and TC83/E1ID-syn. (TIF) [file ppat.1012179.s010.tif]

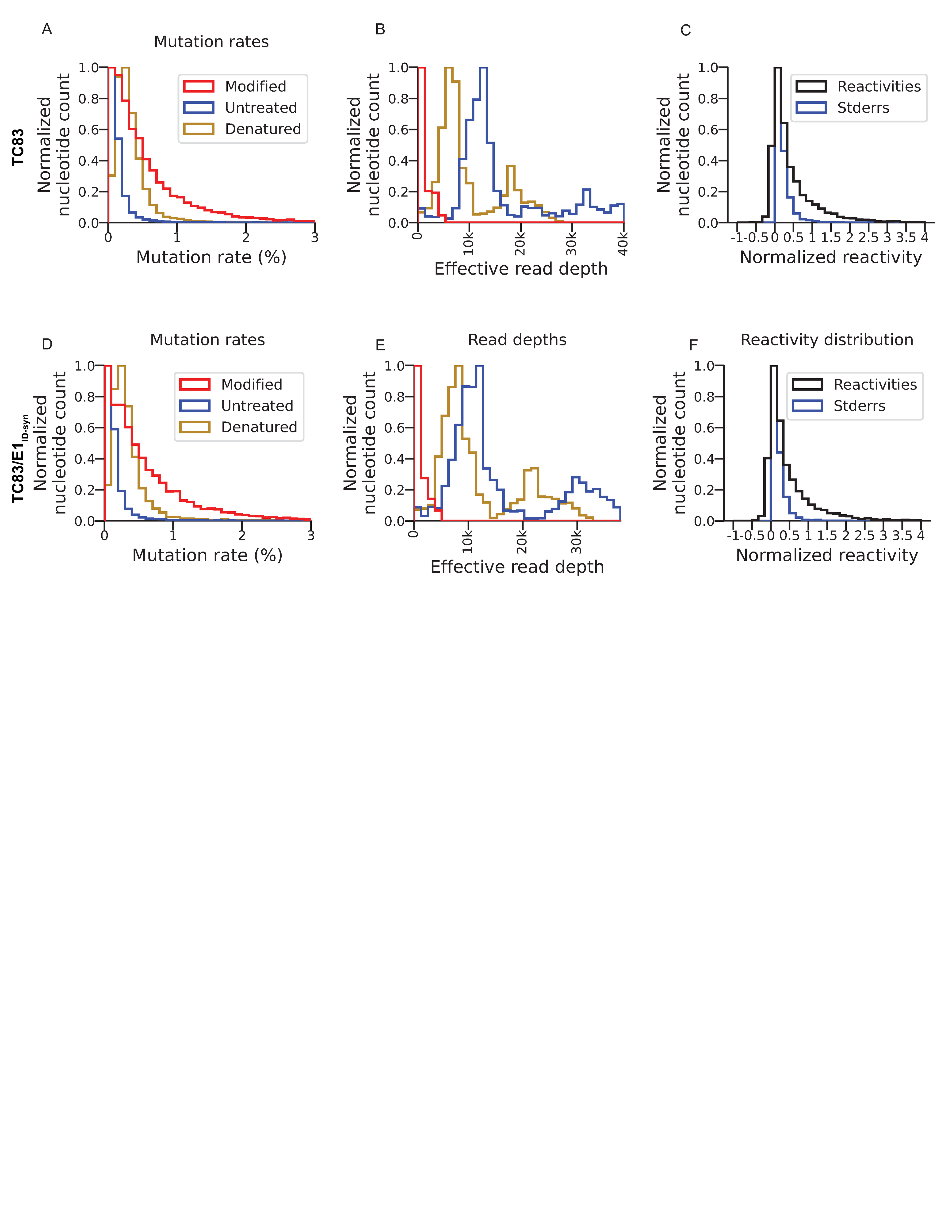

Supplement: S7 Fig — Mutation rates for modified, untreated and denatured control (A) TC83 and D. TC83/E1ID-syn. Read depths for modified, untreated and denatured control (B) TC83 and E. TC83/E1ID-syn. The distribution of the SHAPE-MaP reactivities and the standard error of the reads for C. TC83 and F. TC83/E1ID-syn. (TIF) [file ppat.1012179.s011.tif]

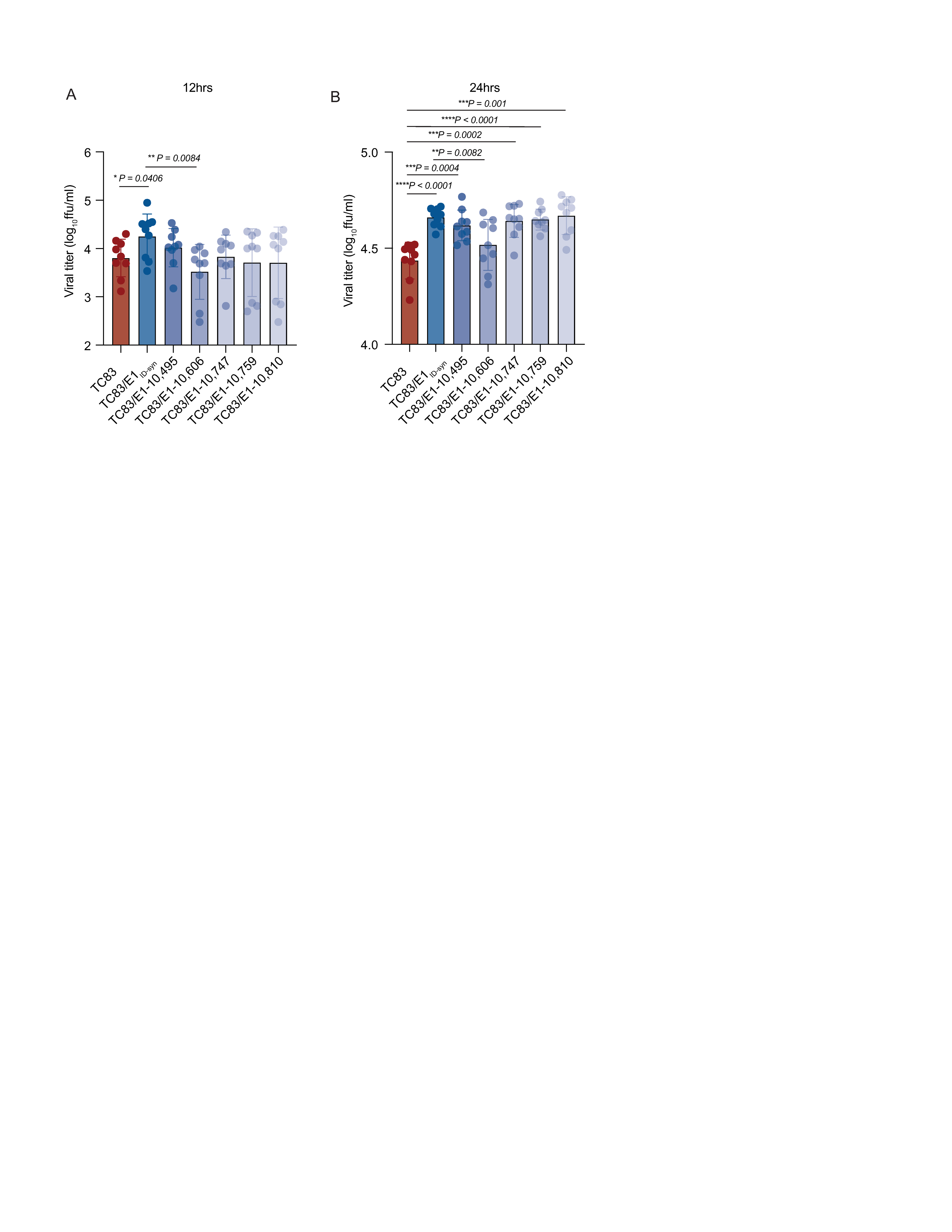

Supplement: S8 Fig — Mutant viruses were made containing a single point mutation from 307537 in the TC83 backbone. Raw264.7 were infected with single point mutants (MOI 0.1) and supernatants were harvested at 12 (A) or 24 (B) hpi and infectious virus titered by focus forming assay (FFA). Each experiment was performed in triplicate three times independently and the mean and SD graphed. Statistical analysis was performed using an unpaired T-test. (TIF) [file ppat.1012179.s012.tif]

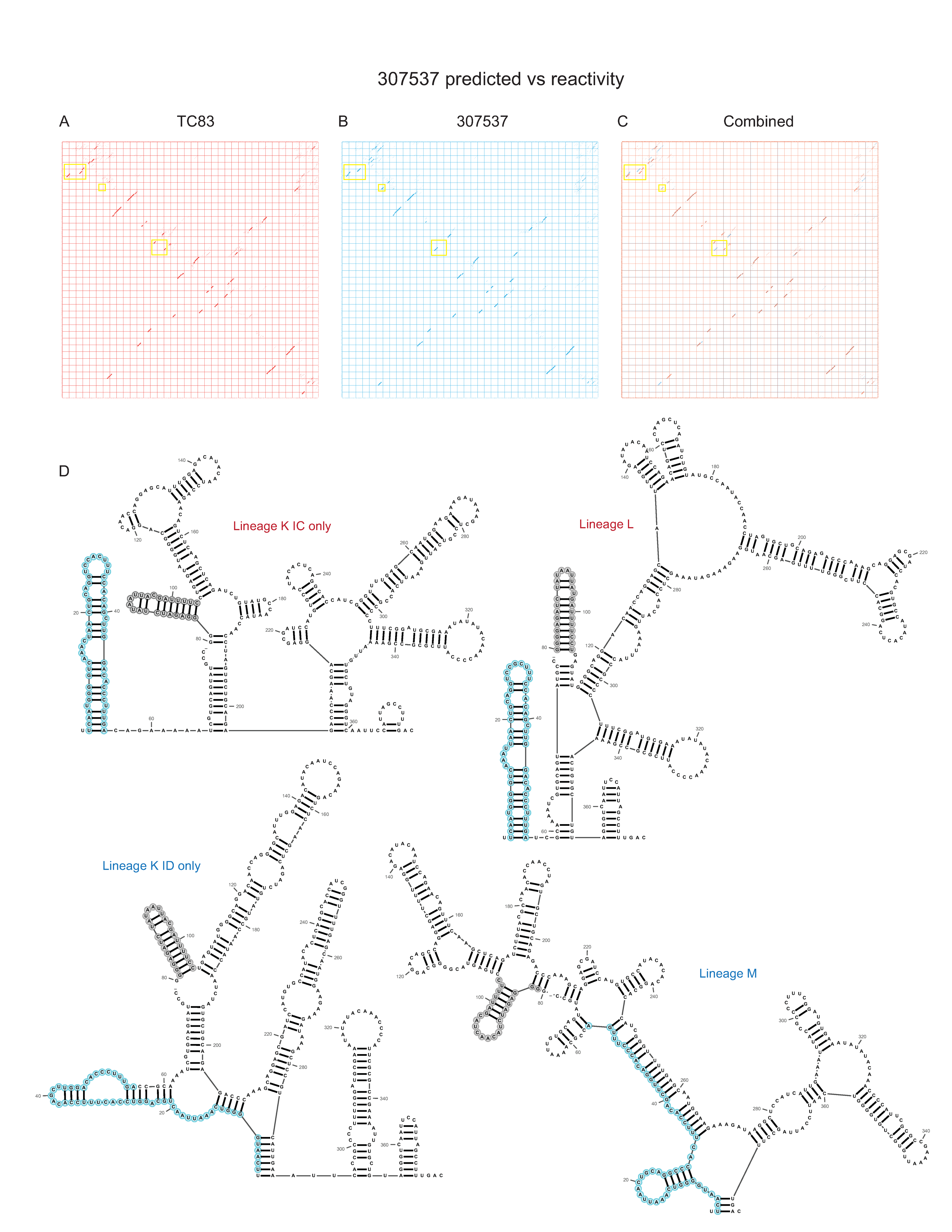

Supplement: S10 Fig — Dot plots from RNAfold [17] predictions of individual SNPs within the E1 core region (10,466–10,843) for (A) TC83, (B) TC83/E1ID-syn and (C) overlayed dotblots. Yellow boxes highlight regions with differences in RNA structure predictions. (D) Predicted RNA secondary structures from RNA alignfold [51]of sequences from lineages K (divided into epizootic IC and enzootic ID), L and M. Steml-loop conserved in epizootic lineages is highlighted in blue, and stem-loop conserved in all lineage K and L highlighted in grey. (TIF) [file ppat.1012179.s014.tif]

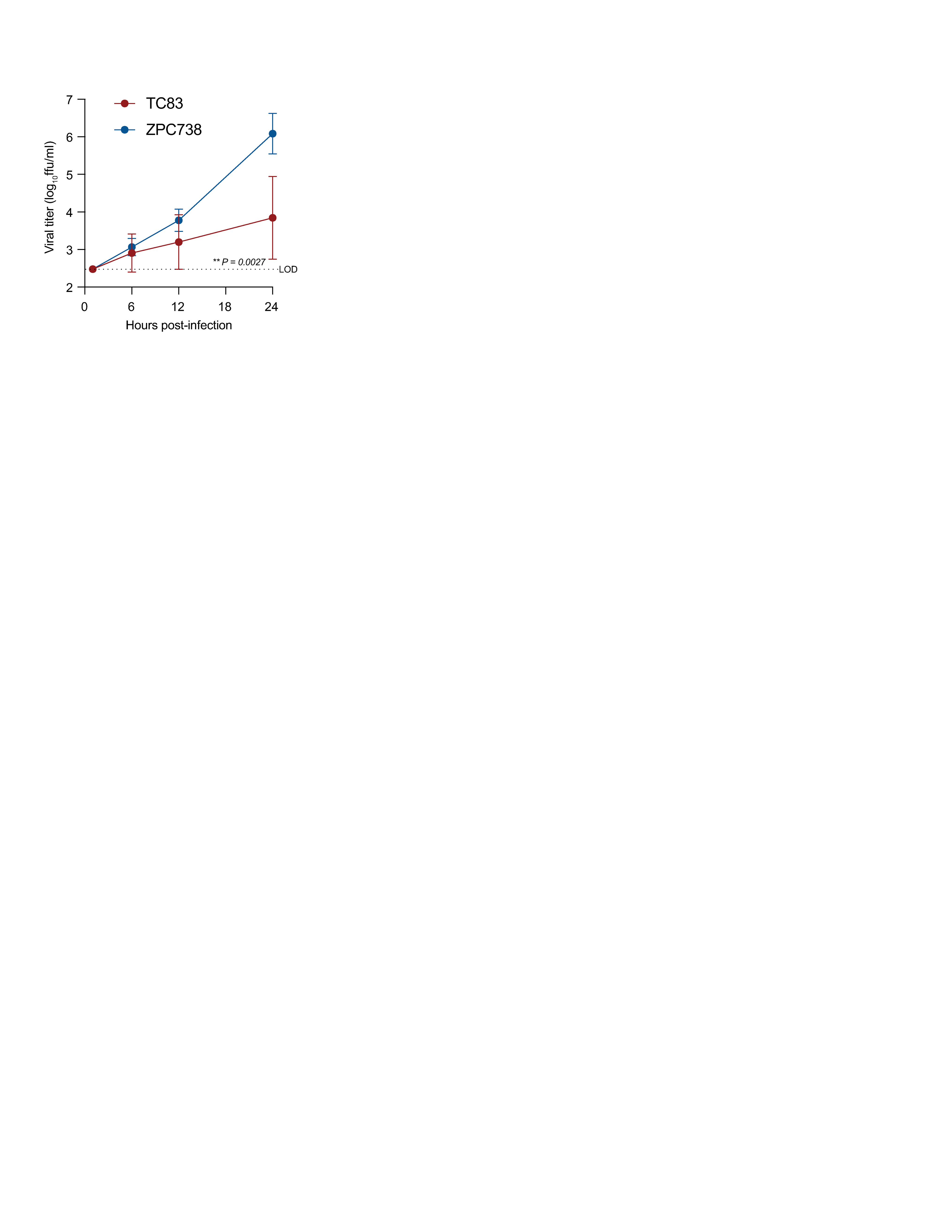

Supplement: S11 Fig — Replication kinetics of VEEV TC83 and ZPC738 in Raw264.7. Cells were infected with indicated viruses at a MOI of 0.1. Cell culture supernatant was serially harvested at 1, 6, 12, and 24 hpi and infectious virus was titered using focus forming assay (FFA). The experiment was performed in triplicate, three independently and the mean and SD are graphed. Statistical analysis was performed by calculating the area under the curve (AUC) for each replicate, and the AUC values from TC83 and ZPC738 were analyzed by unpaired t-test. (TIF) [file ppat.1012179.s015.tif]

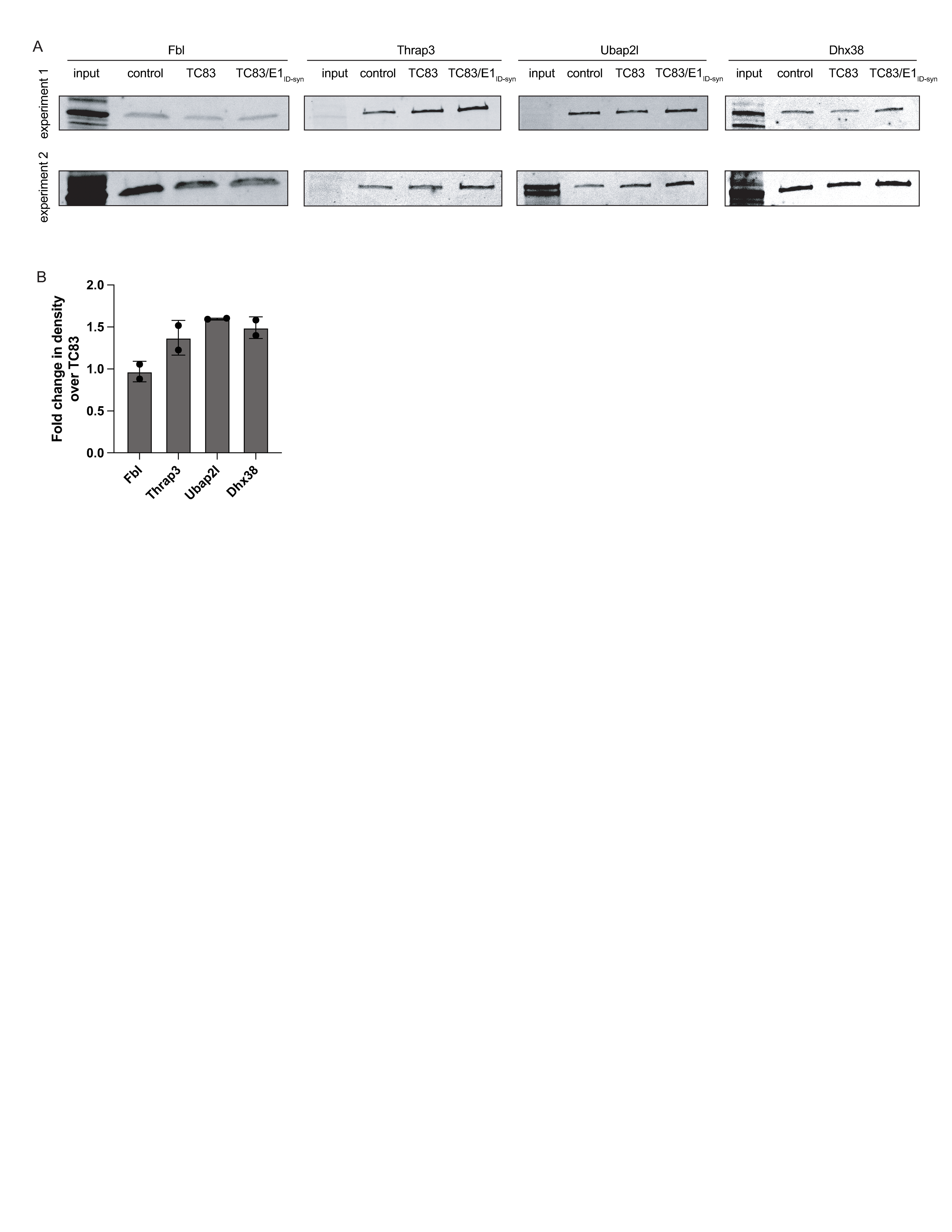

Supplement: S12 Fig — In vitro RNAs encoding 4x repeats of the S1m-aptamer followed by the core region of E1 (10,516–10,808) from TC83 or TC83/E1ID-syn were generated along with a 4x S1m-aptamer only control RNA. RNA was bound to streptavidin beads then incubated with cellular lysates from Raw264.7, and bound proteins eluted and analyzed. (A) Western blot analysis of Fbl, Thrap3, Ubap2l and Dhx38 is displayed for the input lysate control, RNA aptamer control, TC83 or TC83/E1ID-syn aptamer RNAs. (B) Densitometry was performed on the bands using Adobe Photoshop and the fold change in density of the TC83/E1ID-syn over the TC83 RNA aptamer is displayed. This is representative of two independent repeats. (TIF) [file ppat.1012179.s016.tif]
